# Combined Mass Spectrometry and Histopathology Imaging for Perioperative Tissue Assessment in Cancer Surgery

**DOI:** 10.3390/jimaging7100203

**Published:** 2021-10-04

**Authors:** Laura Connolly, Amoon Jamzad, Martin Kaufmann, Catriona E. Farquharson, Kevin Ren, John F. Rudan, Gabor Fichtinger, Parvin Mousavi

**Affiliations:** 1 School of Computing, Queen’s University, Kingston, ON K7L 3N6, Canada; a.jamzad@queensu.ca (A.J.); catriona.e.farquharson.23@dartmouth.edu (C.E.F.); fichting@queensu.ca (G.F.); mousavi@queensu.ca (P.M.); 2 Department of Surgery, Queen’s University, Kingston, ON K7L 3N6, Canada; martin.kaufmann@queensu.ca (M.K.); john.rudan@kingstonhsc.ca (J.F.R.); 3 Department of Pathology and Molecular Medicine, Queen’s University, Kingston, ON K7L 3N6, Canada; kevin.ren@kingstonhsc.ca

**Keywords:** perioperative imaging, DESI, deep learning, deformable image registration, prostate cancer, diagnosis

## Abstract

Mass spectrometry is an effective imaging tool for evaluating biological tissue to detect cancer. With the assistance of deep learning, this technology can be used as a perioperative tissue assessment tool that will facilitate informed surgical decisions. To achieve such a system requires the development of a database of mass spectrometry signals and their corresponding pathology labels. Assigning correct labels, in turn, necessitates precise spatial registration of histopathology and mass spectrometry data. This is a challenging task due to the domain differences and noisy nature of images. In this study, we create a registration framework for mass spectrometry and pathology images as a contribution to the development of perioperative tissue assessment. In doing so, we explore two opportunities in deep learning for medical image registration, namely, unsupervised, multi-modal deformable image registration and evaluation of the registration. We test this system on prostate needle biopsy cores that were imaged with desorption electrospray ionization mass spectrometry (DESI) and show that we can successfully register DESI and histology images to achieve accurate alignment and, consequently, labelling for future training. This automation is expected to improve the efficiency and development of a deep learning architecture that will benefit the use of mass spectrometry imaging for cancer diagnosis.

## 1. Introduction

**Figure 1:**
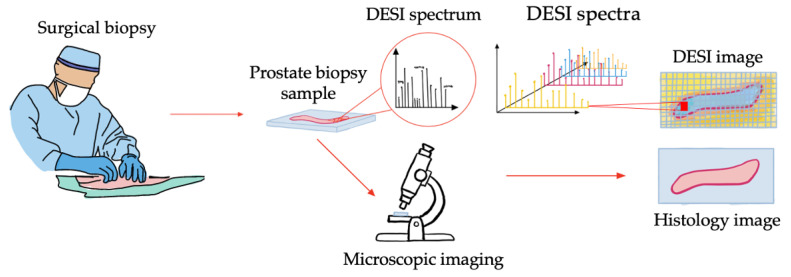
Visual representation of 3D DESI images in a surgical workflow.

Recently, mass spectrometry imaging has gained attention in cancer research owing to its ability to capture the spatial distribution of tissue metabolites, mostly in the context of cancer pathology. Cancer biomarkers can be characterized in relation to tissue pathologies, as well as tumor aggressiveness using this technique [1]. The application of mass spectrometry imaging systems has the potential for perioperative tissue assessment to improve surgical outcomes. Desorption electrospray ionization mass spectrometry imaging (DESI) is an example of a mass spectrometry imaging system that can be used in this application. DESI is an ambient ionization technique that can profile the metabolic signature of tissue samples at a resolution of 50 μm with minimal tissue preparation [1]. The acquired large, multiplexed images can then be compared with gold standard histopathology annotations for investigating their correlation. Figure 1 demonstrates the general concept of DESI data in comparison to pathology imaging.

Should the metabolomic signatures from these images prove to be sensitive and specific in determining the presence or absence of cancer, DESI will be of clinical significance. The current standard-of-care, diagnostic pathology of tissue biopsies, relies on images and their interpretation from classic staining like Hematoxylin and Eosin (H&E) to complex immunohistopathology. In comparison, DESI imaging fortified by advanced analysis can be more time efficient and objective. Additionally, DESI requires minimal sample preparation and is not destructive which allows for direct pathology annotation on the same tissue slide [1].

Developing a perioperative cancer evaluation system is dependent on the creation of a large database, to train models that precisely associate the metabolomics signature with histopathology characteristics in a pixel-based manner. One of the difficulties faced in developing this perioperative system is that images taken of the same tissue sample with mass spectrometry imaging and histology are not always perfectly aligned. In the case of DESI imaging this is usually due to the presence of noise during data acquisition or tissue smear during staining. Subsequent analysis of the data in the presence of such noise is challenging, especially for tissues with high heterogeneity like the prostate. Therefore, to collate a robust labelled dataset, an efficient registration approach that aligns mass spectrometry images to their corresponding histology must be devised. In this study we consider this as multi-modal registration because the end goal is to register a 2D representation of a DESI image to its corresponding histology.

Registration, the process of aligning two or more images, is a well-established area of medical image analysis that has been focus of a lot of early and continuing research [2]. Registration methods can be categorized by several different criteria, one of which is the nature of the transformation: rigid, affine, projective, or curved [2,3]. Curved registration is commonly referred to as deformable image registration, which is of particular interest for DESI imaging because multi-modal data acquisition can lead to deformities in images of the same tissue sample. Addressing these deformities is necessary to avoid losing information, as this is still a new imaging technique and access to data is limited.

Conventional deformable registration approaches include intensity-based or feature-based algorithms [3]. The general goal of intensity-based methods is to iteratively search for the transformation that best aligns two images by optimizing a cost function [4]. Feature-based methods, however, rely on feature segmentations from each image, that are used to optimize a cost function to predict a transformation based on feature correspondence [4]. Of these two approaches, intensity-based methods are generally favored because they do not require image segmentations or annotations [4]. Advanced Neuroimaging Tools (ANTS), Elastix and NiftyReg are examples of well-established, intensity-based registration techniques that have been demonstrated and applied to various medical imaging problems successfully [5,6,7]. However, as these approaches are all reliant on iterative optimization, the field of deformable registration has moved towards deep learning (DL) to improve the computational efficiency of this process.

Recent literature have shown that DL can improve computation cost by using a learned optimization strategy in place of iteration [8,9,10,11]. The DL approaches that are used for deformable registration are either supervised or unsupervised, indicating that the models are either trained with or without ground truth registrations [8]. One of the benefits of using an unsupervised approach is that it eliminates the requirement of performing manual registrations as a baseline [12,13]. An example of a frequently used unsupervised, deformable registration architecture is VoxelMorph by Balakrishnan et al. which was initially developed to register unimodal MR brain images and has since been applied to several other medical image registration problems [14,15,16]. This architecture performs comparibly to the aforementioned traditional techniques while improving runtime significantly [14,17]. There has however, been minimal investigation of VoxelMorph, and unsupervised registration networks in general, for multi-modal registration problems [8,18].

Another important part of developing a database of labelled mass spectrometry images is the ability to quantify the success of histopathology registration for label confidence. To evaluate registration methods, several metrics are considered in the literature, including mutual information, sum of square distance and fiducial registration error (FRE) [4,19]. These metrics can be used to capture both the general alignment of two images and the registration accuracy of high resolution features [8]. Traditional approaches to calculating these metrics face the same challenges as manual registration (exhaustive operator intervention, time intensive, high cost, etc.). Because of this, DL methods have also been proposed for performing these evaluations [20,21,22,23]. However, DL-based evaluation strategies are all supervised and heavily reliant on access to large manually annotated datasets which makes it difficult to apply them to emerging imaging techniques [24].

In this paper, we present MassReg, a DL architecture for registration of mass spectrometry images to their corresponding digital histology as a step towards perioperative tissue assessment for cancer surgery. We use DESI imaging as an example of a mass spectrometry imaging technique for validation of this new registration approach. In doing so, we address two under-investigated aspects of this research domain, namely unsupervised, multi-modal registration and evaluation of registration in the absence of a large manually annotated dataset [8]. In addition to this architecture, we also present new approaches for data simulation, augmentation, and iterative training to further avoid dependency on a large database of manual annotations that is not yet available for DESI imaging. Due to the importance of prostate cancer as the most prevalent cancer among Canadian men and the third leading cause of male cancer related death, we validate the proposed approach on cross-sections of prostate biopsy cores [25].

## 2. Materials and Methods

**Figure 2:**
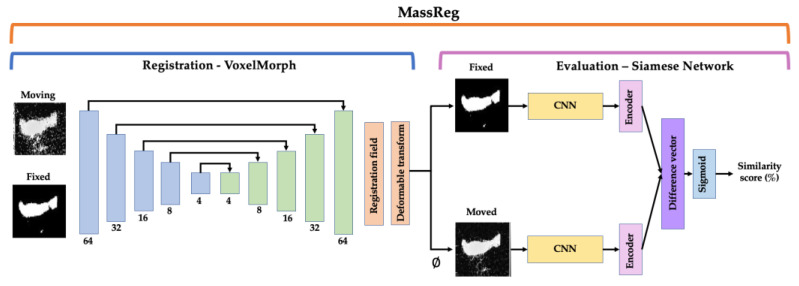
Overall network architecture for MassReg. Left half—VoxelMorph framework for registration. This network is based on a U-Net which is an encoder/decoder network with skip connections. Right half—Siamese network for evaluation. This architecture consists of two identical convolutional neural networks (CNNs), that feed into two encoders, a difference vector and finally a sigmoid activation function. The input to the network is a DESI/histology image pair and the output is the registered image pair and a similarity score.

The following sections detail the implementation of our MassReg network and testing strategy. We categorize the network into two parts: the first half, which performs registration and the second half, which evaluates the output of this registration (Figure 2). For registration, we use an unsupervised deformable approach and train the network in two iterations with simulated and real image pairs. For evaluation, we implement a Siamese network and train the network in multiple iterations on both real and simulated data. We test our proposed method on DESI and histology image pairs from prostate biopsy cores and compare our results to previously established registration methods and evaluation metrics.

### 2.1. Network

The network we propose for this perioperative system, MassReg, is comprised of two DL architectures that are used to perform both image registration and registration evaluation. The overall network architecture is described in Figure 2.

The first half of the network is used for registration and is based on a framework for unsupervised, deformable registration called VoxelMorph [14]. This approach takes two images as inputs (fixed and moving), and outputs a transformation field that can be applied to the moving image for registration. The learning structure is based on a U-Net which consists of an encoder network and a decoder network with skip connections. The original VoxelMorph paper provides more detail on this CNN architecture [14]. We extend the original approach by applying it to a multi-modal registration problem and introducing pre-training which is discussed in more detail in Section 2.6. We chose a deformable image registration approach for this application because of the nature of DESI and histopathology data collection. In many cases, the prostate cores were deformed during staining which led to inconsistent contour shapes for registration. To preserve as many data points as possible it’s important that we can still accurately validate cross sections of the tissues that may have been deformed at this stage.

The second half of the network is a Siamese network which is used to evaluate the output of the first half. The network structure that we implemented is derived from Koch et al. where each branch of the network consists of two identical convolutional neural networks (CNN). These CNNs feed into two separate encoders which are then connected and activated by a dense layer with a sigmoid activation function [26,27,28]. If the feature vector from each encoder is the same or similar, the two images are considered registered; if it is different, the two images are considered unregistered. One of the advantages of Siamese networks is that they can facilitate one-shot learning which requires minimal training data. This application is similar to the study presented by Fu et al. who suggested a Siamese network architecture for automatic landmark pair detection in 4DCT lung images [20]. However, while their approach is dependent on a large dataset of manually, annotated images, we make use of data augmentation and simulation instead.

In summary, MassReg can be used to register two multi-modal images and assign a similarity score (as a percentage) that reflects the success of the registration. One of the benefits of this implementation is that expressing the outcome of the registration as a percentage requires no prior knowledge about the domain and standard evaluation metrics. Moreover, as we make use of simulation and augmentation strategies, we are not limited by the size of our dataset or lack of manual annotations.

### 2.2. Dataset

The dataset used includes 33 prostate biopsy cores from 19 patients, age 56–74, sampled with a needle biopsy gun after radical prostatectomies [27]. After the cores were taken from the prostate gland, they were frozen in optimal cutting temperature (OCT) compound, cryosectioned and fixed on a microscope for further analysis. These samples were then stored at −80° for 3 months.

For metabolic analysis, a DESI scan was taken of each tissue sample using a 2D DESI source and the Xevo-G2 XS Q-Tof mass spectrometer (Waters Limited, Milford, MA, USA). DESI imaging involves infusing a solvent stream onto the tissue sample at an angle to propel analytes from the surface of the tissue to the mass spectrometer. A solution made of up 95% methanol and 5% water and leucine enkephalin was used as the solvent. The solvent spray was set at an incident angle of 75° and a height of approximately 0.5 cm, while the inlet to the spectrometer was at a height of approximately 1 cm from the tissue sample. The specific DESI source parameters from this acquisition are available in Morse et al. [27]. Following this, the slide was processed with H&E stain and scanned before undergoing histology analysis. The spatially recombined DESI image and corresponding histology image were considered image pairs for this study. In total, the dataset consisted of 33 image pairs, 25 of which were used to train the network while the remaining 8 were used for testing in a 10-fold cross validation scheme. The patients in each training and test fold were mutually exclusive.

### 2.3. Preprocessing

**Figure 3:**
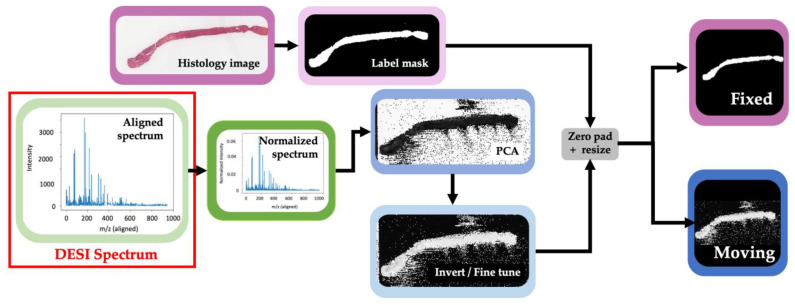
Full preprocessing pipeline. Top row demonstrates the steps for processing the histology images. Bottom row demonstrates the process for representing DESI scans as a 2D image. Output on the right is the final two images used for registration. Histology images are considered as fixed while DESI data is considered as moving.

The full preprocessing pipeline is shown in Figure 3. The histology images of each prostate core were converted to a binary label mask by applying color space conversion and thresholding between the stain on the tissue and the slide (background). In this new representation, a value of 1 indicates that the pixel represented tissue and a value of 0 indicates that the pixel represented background. The image was then zero padded to a square to preserve the aspect ratio before being resized to 64 × 64 pixels. This label mask from each histology image was considered a fixed reference for registration.

For DESI images, each pixel represents a spectrum that was taken at a specific spatial cross section on the tissue slide, Figure 3 demonstrates what this spectrum looks like. In a DESI spectrum, the x-axis represents the relationship between the mass of an ion and the number of charges it carries (*m*/*z*), while the y-axis represents the intensity of that ion signal in the corresponding cross section of the tissue. Before calibration, the pixels of these images are not directly comparable because the spectra may be misaligned due to instrumentation drift over time. A technique called lock mass calibration was performed to ensure that the peak value for each spectrum corresponds to the correct mass to charge ratio. This is a common approach for calibrating mass spectrometry instruments where a compound of known mass is included in each sample as an internal standard [28]. This is important to ensure that we are performing accurate molecular comparison. Each spectrum was then normalized by its total ion current, which represents the summed intensity across the entire spectra [29]. After normalization, each spectrum or pixel in the DESI image contains 900 *m*/*z* peaks. To reduce the dimensionality of the data for visualization, principal component analysis (PCA) was used to go from these 900 features to 3 for each pixel. We found that reducing the image to 3 components was sufficient for capturing the contrast necessary to obtain a clear image of the tissue contour. This is because we assume that the first principal component, which is representative of the highest variance in the data, is caused by the variance between sampling the tissue versus the background slide. However, in some cases this is better represented by the second or third principal component, so it is better to use all 3. Each image was then individually fine-tuned by selecting the principal component that could best separate tissue from background and then applying varying thresholds to obtain a clear image of the tissue contour. For samples where the PCA representation resulted in a white background and black tissue contour, the images were inverted. Finally, a threshold was applied to the images so that the maximum intensity value of the images is 1, zero padded and resized to 64 × 64 as before. The resulting image was then considered the moving reference for registration.

At this stage, the DESI image and histology mask can both be considered label masks although in practice, the histology masking would be reversed after registration for histological analysis. For this reason, we consider this a multi-modal image registration problem.

### 2.4. Augmentation

As the dataset for this project is limited, data augmentation was performed to increase the size of our training set. This augmentation was performing by applying rotations of 45°, 90°, 180°, and 270°, in addition to mirroring the images prior to and following rotation. Each histology mask and DESI image were rotated in a similar way so that the pair are always matching. During data augmentation, we ensured that the entire biopsy core is always in the image frame. In total, the size of the training data set was increased from 25 to 250 pairs. To address the limited dataset size (33 unique prostate cores), the following experiments were performed using 10-fold cross validation, where augmentation was only applied to the training fold for each repetition.

### 2.5. Data Simulation

Beyond augmentation, simulation was performed to create data that mimics DESI and histology images pairs. The purpose of this simulation was to train the network to separate the background noise that is present in DESI images, from tissue during both registration and evaluation.

**Figure 4:**
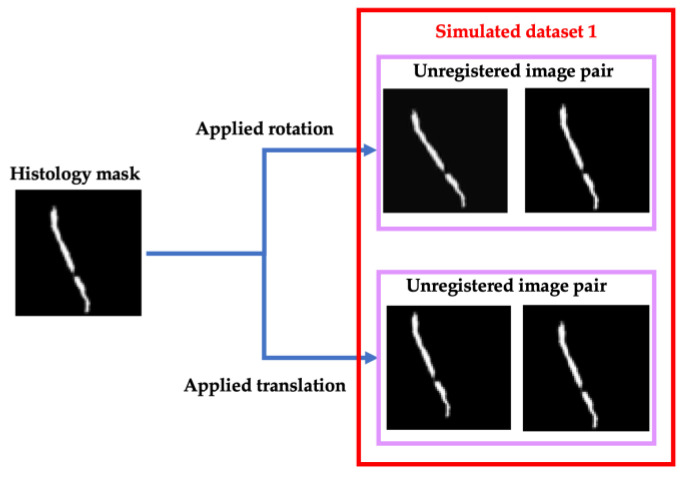
Examples of simulated data process for generating misaligned, unimodal image pairs.

For registration, a simulated training set was generated from the augmented histology masks to teach the network to correct small rotational and translational misalignment. This misalignment was defined as rotations between 3° and 10° and translations up to 12 pixels in one direction. The masks were modified by applying this misalignment to create an un-registered image pair (Figure 4). To assign a similarity score to these simulated un-registered pairs, the score was distributed based on the extent of the applied transformation. If the augmented mask was rotated 3° the corresponding pair was assigned a similarity score of 0.875 versus if it was rotated 10°, the pair was assigned a score of 0. Similarly for translation, if the augmented mask was translated 2 pixels, the pair achieved a similarity score of 0.8 versus if it was translated 10 pixels it was assigned a score of 0. All these scores were assigned in a linear distribution with even increments, meaning the most significant applied transformation received a score of 0, and the score was increased by the same amount as the applied transformation decreased. Images with no applied transformation received a score of 1.

**Figure 5:**
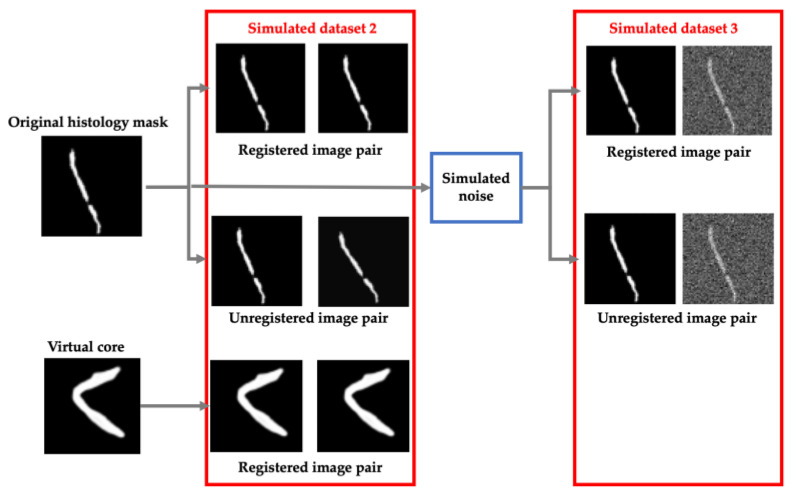
Left—Data simulation approach for generating unimodal and virtual registered and unregistered image pairs. Right—Data simulation approach for generating multi-modal registered and unregistered image pairs.

For evaluation, data simulation was approached in two additional ways: adding shape diversity for contour registration and simulating background noise that resembles multi-modal image registration. For this half of the network, two different simulated datasets were generated (Figure 5).

The first simulated dataset consisted of histology cores that are rotated and translated as before to simulated unregistered image pairs as well as identical copies of the histology masks to simulate registered pairs. Additionally, we added simulated successful registrations from 10 virtual biopsy cores to introduce diversity in our training set. These virtual cores were generated manually by drawing prostate cores in unique configurations. These images were then augmented as before and copied to simulate 100 more successful registration pairs. This process is shown in more detail in Figure 5 (Simulated dataset 2).

To simulate multi-modal registrations, random Gaussian, periodic and Perlin noise was added to each histology mask which were then aligned or misaligned as before. The outcome of this process is shown in Figure 5 as well (Simulated dataset 3).

### 2.6. Experiments 

**Figure 6:**
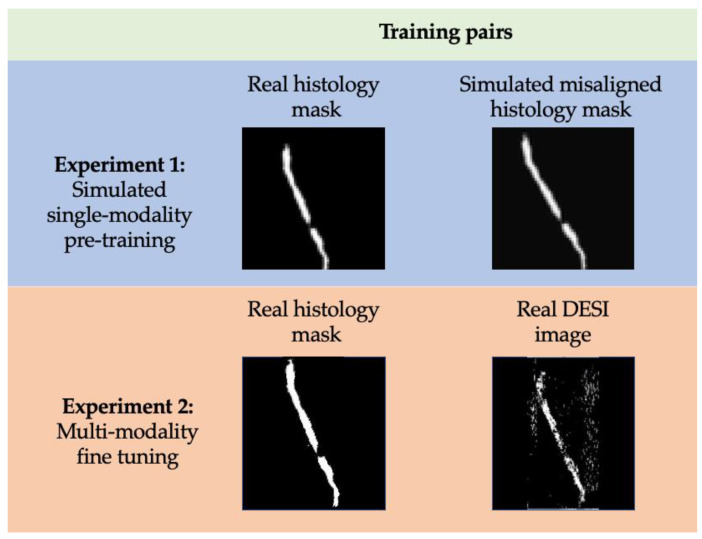
Definition of training pairs used for experiment 1 and experiment 2 of training the first half of the network. In each training pair the simulated or DESI image is considered moving while the real histology mask is considered fixed.

Registration: For registration, the first half of the network was trained in multiple successive, increasingly more complex steps. These steps are described as different training experiments (Figure 6). Experiment 1 was performed with simulated image pairs, while Experiment 2 was trained on real image pairs. Figure 6 describes this distinction in more detail.

The goal of Experiment 1 was to teach the network to recognize and correct rigid registration errors between unimodal images that are misaligned. In doing so, we pre-trained the network to concentrate on registration of tissue contours before introducing a second imaging modality. Simulated dataset 1, which consists of unregistered (translated and rotated) image pairs was used for this pre-training. The VoxelMorph framework was trained on these images for 20 epochs with a batch size of 11. An Adam optimization function was used alongside mean squared error (MSE) and l2 loss functions.

The network was then fine-tuned for multi-modal registration in Experiment 2. This training experiment was performed on the real, unregistered DESI and histology image pairs. With this data, the network was trained for 30 more epochs with a batch size of 11. The same optimization and loss functions were used for both pre-training and fine-tuning. After each experiment, we tested the output of the network on real, DESI and histology image pairs that were held back for testing.

Evaluation: For evaluation, we implemented a Siamese network that is also trained on simulated data as well as real, manually registered image pairs. Again, we trained the network in multiple, increasingly more complex steps, which are considered training experiments [26]. Adam optimization and binary cross entropy loss are also used to train the network.

**Figure 7:**
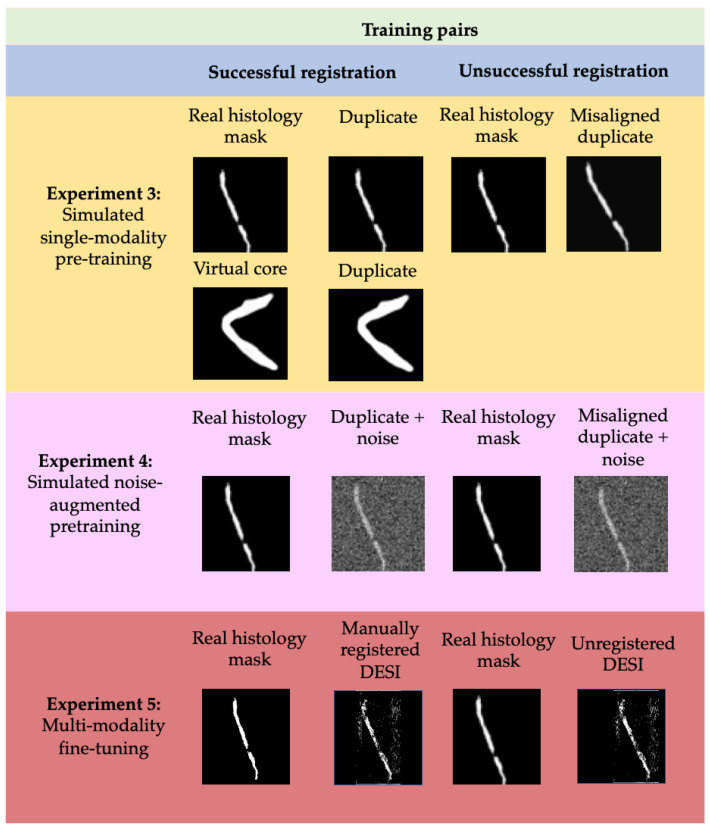
Training pairs used for training the second half of the network iteratively. Both successful and unsuccessful registrations are considered.

The training process consisted of three experiments where simulated and real data is used to realize successful and unsuccessful registrations. Following each training experiment, the weights of the network were preserved so that the process was not restarted. Figure 7 describes the distinction of training data used for each experiment in more detail.

Experiment 3 was performed on simulated noise-free image pairs and virtual cores from simulated dataset 2. Duplicates of the histology masks/virtual cores are considered successful registrations and labelled 1, while misaligned histology masks are considered unsuccessful registrations and labelled 0. The goal of this experiment was to teach the Siamese network to associate misalignment of the central core with a low similarity score and conversely, alignment of the central core with a high similarity score.

Experiment 4 was performed on simulated image pairs from simulated dataset 3. At this iteration, duplicates of the histology masks with added noise are considered successful registrations, while misaligned duplicates with added noise are considered unsuccessful. The goal of this experiment was to teach the network to ignore background noise and quantify similarity by looking exclusively at the alignment and shape of the central contour.

The fifth and final experiment was performed on manual registrations of DESI and histology masks. These manual registrations were performed using Fiducial Registration Wizard, one of the Slicer image-guided therapy (IGT) modules (www.slicerigt.org (accessed on 9 September 2021)) in the open-source medical imaging platform, 3D Slicer [30]. The mean fiducial registration error from these manual registrations was determined to be less than 50 microns which indicates that they should correspond to a high similarity score. Therefore, these manual registrations are considered successful and labelled as 1 whereas the original, unregistered image pairs are considered unsuccessful and labelled as 0.

Each training experiment was performed for 2000 epochs with a batch size of 32. In each round about 70% of the training data was used as the training set and the remaining data was used for validation. The validation data was used every 200 epochs by performing 20-way one shot learning [26]. This is a technique for monitoring training progress where an image was compared to 20 images, 19 that do not represent its corresponding registered image pair and 1 that does, and the accuracy of this prediction is recorded. The results of this one-shot learning on the validation data were used to inform the decision to train for 2000 epochs in each round.

The total number of image pairs used in each training experiment for both the first and second half of the network is described in Table 1.

### 2.7. Ablation Studies

Having established the methods used for implementation and training, the following subsections outline the processes used to evaluate the sensitivity of the MassReg network. We approach this by testing the network response to applied misalignment, computing the tissue volume overlap during registration and reporting a baseline evaluation metric for each test fold.

Affine registration comparison: For other imaging techniques, a simple affine registration approach may be of interest. Therefore, we also tested our pre-training approach on an affine registration network. For affine registration we applied a technique that uses a CNN to predict a vector of length 6 that details the parameters for controlling image rotation, scaling, translation, and shearing, followed by a spatial transformer that applies the transformation to the moving image [31,32]. Th approach has had demonstrated success on the MNIST dataset. The network consists of three parts: the localization network, the grid generator, and the sampler. For any given input, the network determines the transformation that should be applied to the image, creates a sampling grid from the predicted transformation and then produces the output vector. We used normalized cross correlation (NCC) loss and optimized the network with stochastic gradient descent (SGD). The exact same pre-training approach was applied to this new network with the same cross validation data and the same number of epochs for each round, so we could conduct a direct comparison to VoxelMorph.

Sensitivity to rotation and translation: To validate the similarity scores assigned by the Siamese network, a sensitivity test was performed on simulated registration data. Histology masks from a test set from one of the cross-validation folds (that the network had not seen before) were modified in various ways to detect the network’s sensitivity to applied rotation and translation. After applying a modification (rotating/translating the image) the new image was considered the registration pair for the original mask and the network was used to predict a similarity score. Rotation was varied between 3° and 12°, and translation between 0 and 10 pixels in both the x and y direction. The resulting similarity scores from each of the 8 modified histology masks were averaged to evaluate the network response.

Tissue overlap analysis: To analyze the first half of our network, we compute the dice score, which is a measure of volume overlap, at each stage of training. Dice score is computed using,

(1) $$DS=2\;\cdot \;\frac{Im_{fixed}\;\cap \;Im_{transformed}}{Im_{fixed}+Im_{transformed}}$$

where $Im_{fixed}$ is the segmentation of the fixed image (histology mask), and $Im_{transformed}$ is the segmentation of the transformed image (registered DESI image). A dice score of 1 indicates that two images are completely overlapping while a dice score of 0 indicates that there are no pixels in common. Because we do not have segmentations of what is background noise versus tissue in the DESI images, any pixel that had an intensity above 0 in the DESI images is considered tissue and labelled 1 for comparison. Although this means we are likely considering background noise as tissue in some cases, it gives us a general idea of the overlap of the central tissue contour which is an indicator of the success of the registration. Additionally, we use dice score to monitor the progress of our registration result during each training experiment.

For comparison with a baseline method, we also perform b-spline registration with the Elastix toolkit and compute the dice score of the resulting registrations. This is a common approach for deformable registration that is not reliant on DL. This registration was implemented using the Insight toolkit (https://itk.org (accessed on 9 September 2021)).

Further evaluation metrics: In addition to the sensitivity testing previously described, we analyze the second half of our network by also computing FRE as a baseline evaluation metric for comparison. The FRE of each registration was calculated by measuring the average root mean squared error (RMSE) between 3 corresponding fiducials that were manually selected on each tissue contour in the registered images.

## 3. Results and Discussion

**Figure 8:**
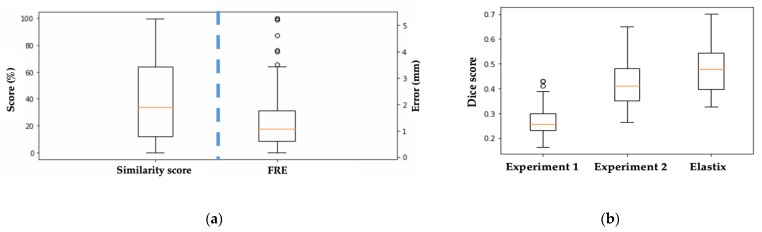
(**a**) Box-whisker plot showing the similarity score spread versus FRE spread for each test image across all 10 cross validation folds. (**b**) Box-whisker plot showing the dice score as a comparison for experiment 1, 2 and Elastix baseline across all 10 cross validation folds.

**Figure 9:**
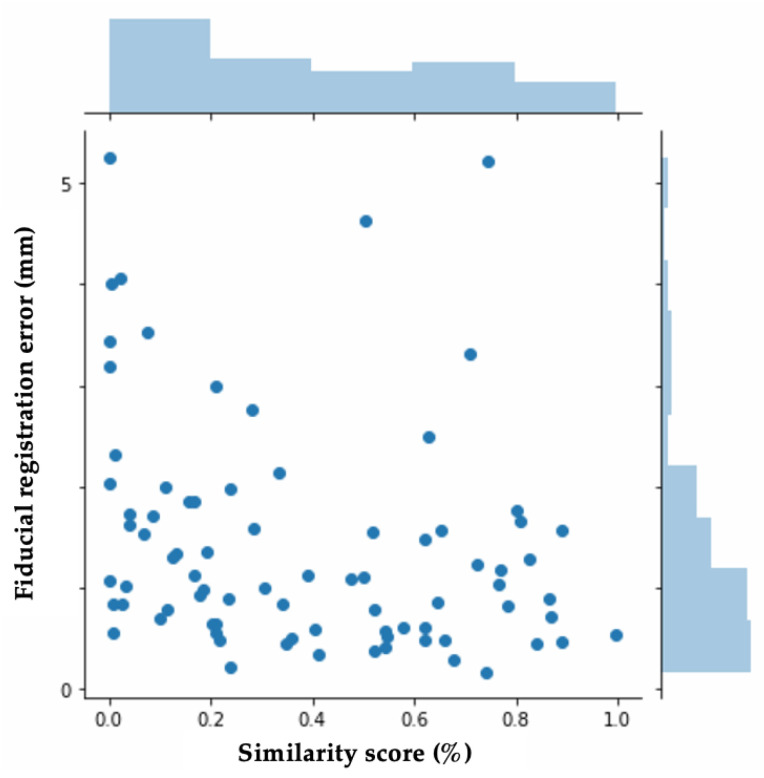
FRE and Similarity score distribution.

As described, the second half of MassReg is used to evaluate the output of the first half. Therefore, the success of the first half of the network is being quantified by the second half during testing. Figure 8a demonstrates the comparison of the average predicted similarity score, which is the evaluation metric assigned by MassReg, and the average FRE for the test images in each fold. Figure 9 also demonstrates this relationship with a histogram.

This histogram demonstrates that the FRE and the similarity score are not closely related. We anticipated this based on the way the FRE was computed for our dataset. As many of the tissue contours do not have multiple distinct landmarks that can be used for manually computing the FRE, this metric is subjective and likely prone to error. This further reiterates our initial motivation to develop a new way of reporting registration accuracy that is not reliant on manual annotation. This histogram is included to provide context as to how this new metric compares to existing ones.

Across all folds, we found a median similarity score of about 33.8% and a median FRE of about 1.1 mm. Additionally, there is a similar distribution of both high FRE values (over 3 mm), and low similarity scores (under 10%). The main discrepancy we came across was that the network would sometimes assign low similarity scores to registrations with significant background noise, making it an inaccurate assessment of the registration result for the central contour. To investigate in more detail, we look at specific examples from each half of the network during various training experiments.

**Figure 10:**
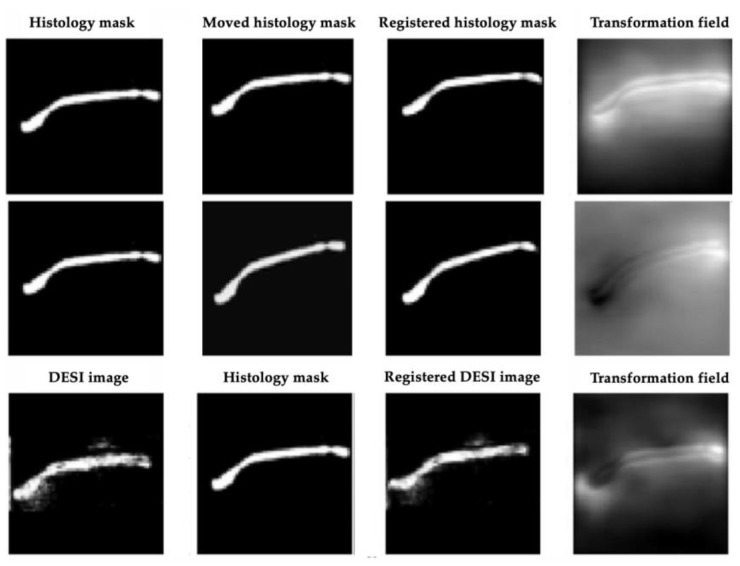
MassReg registration results after training experiment 1. Row 1—Simulated unregistered pair (translation). Row 2—Simulated unregistered pair (rotation). Note—the registered histology mask in column 3 of rows 1 and 2 should be identical to the moved histology mask in column 2, as observed. Row 3—Real unregistered pair. The registered DESI image should be spatially aligned with the histology mask in column 2. In this registration, the DESI image is not moved close enough to the right or shifted enough vertically.

For the first half of the network, two training experiments were performed. After experiment 1, MassReg could successfully correct rigid registration errors on the simulated noise-free image pairs. However, when it was applied to real DESI and histology pairs, the network did not adapt well to background noise. This can be observed in Figure 10. Note that the transformation field represents the applied deformation during registration. We expect to see the contour outlined as most of the registration involves lining up the contour edges. The intensity values of the transformation field correspond to the direction of deformation which is why in some cases the field is light and in some cases it is dark.

**Figure 11:**
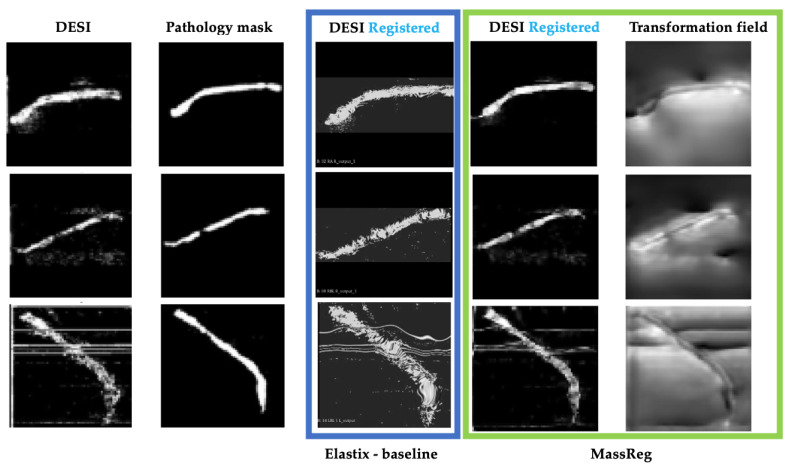
MassReg registration results after experiment 2 of training on real un-registered image pair. Left to right: DESI-MS images, corresponding pathology masks, registered DESI images with Elastix, registered DESI images with MassReg, transformation field from MassReg output.

After Experiment 2, the network was tested again on the real image pairs (Figure 11). By visual inspection, with this additional training, the network was shown to successfully identify the contour of the tissue while ignoring most of the background noise. The output of this experiment also closely resembles the output of the Elastix baseline, although the borders of the MassReg registration are more clearly defined.

We also computed the dice score after Experiments 1, 2 and after using Elastix for registration. In doing so, we found that the dice score increased by approximately 200% in every fold after Experiment 2 (Figure 8b). The average dice score for Elastix is slightly higher than our approach which is reflective of the assumption that background noise is tissue.

These results validate our hypothesis that two training iterations were necessary for correcting rigid and deformable registration error and validate that the VoxelMorph approach (the first half of MassReg) can be used for this multi-modal image registration problem.

**Figure 12:**
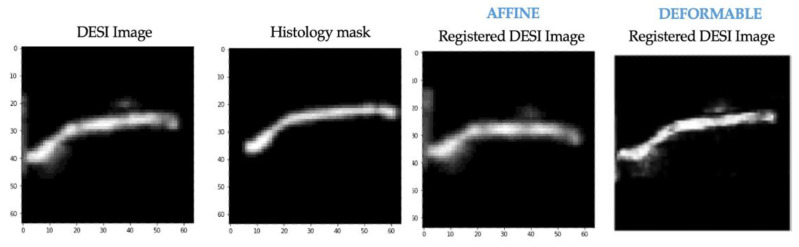
After training experiment 1. Left to right—DESI image (moving), histology masks (fixed), transformed DESI image (moved)—affine transformation, transformed DESI image (moved—deformable transformation.

The same training experiments were performed on the affine registration network that was implemented for comparison. The results after training experiment 1 for this network on the same example from Figure 10 are shown below (Figure 12). At this stage, neither affine nor deformable registration is sufficient. This is apparent based on the misalignment between the histology mask and both registered DESI images.

**Figure 13:**
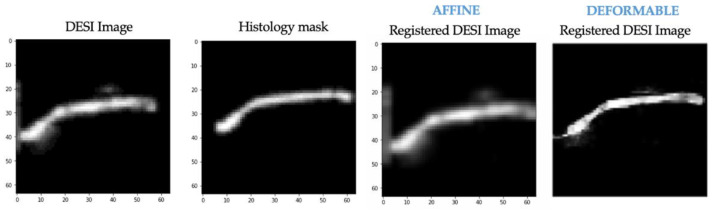
After training experiment 2. Left to right—DESI image (moving), histology masks (fixed), transformed DESI image (moved)—affine transformation, transformed DESI image (moved)—deformable transformation.

We also applied affine registration again after experiment 2 and achieved similar registration results (compared to VoxelMorph) with regard to misalignment correction (Figure 13). This suggests that our pre-training approach can be extended to affine registration. In applications where deformable registration is not ideal, modifying the MassReg network would simply require replacing the first half of the network with this Spatial Transformer network rather than VoxelMorph.

Although Figure 13 suggests that an affine registration network can be used for this image registration problem, more investigation should be done with respect to the training approach and hyperparameters to achieve optimal registration results. For example, the training should emphasize misalignment correction rather than multi-modality fine tuning. We wanted to demonstrate a direct comparison, so these parameters were not modified in this implementation.

**Figure 14:**
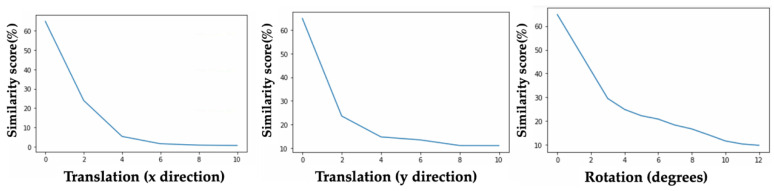
Sensitivity test results for Siamese network. Left—sensitivity to applied translation in the x direction, Middle—sensitivity to applied translation in the y direction, Right—sensitivity to applied rotation.

Next, we evaluated the sensitivity of the second half of MassReg. Figure 14 demonstrates the results of these sensitivity tests on one of the train/test folds, however similar results were observed for each cross-validation fold.

The similarity score predicted by our network is updated as expected during these tests. With both incremental rotation and translation, the similarity score decreases.

**Figure 15:**
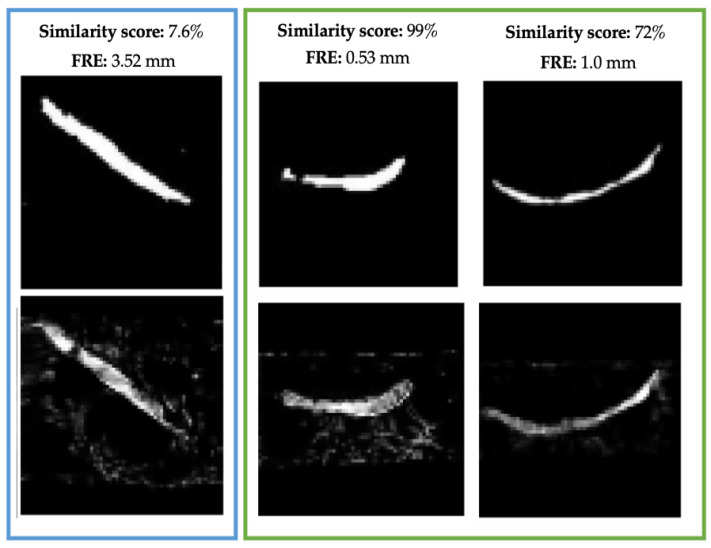
Registered pairs and their corresponding FRE and similarity score from the test set in the first fold. FRE is demonstrated in mm.

Finally, we compared the similarity score assigned by our Siamese network on the DESI and histology image pairs to the FRE of each registration to determine the benefits of employing this method of registration assessment. Figure 15 demonstrates the registration results on three samples from our test sets as well as their corresponding similarity score and FRE. In each case, the similarity score is reflective of the FRE value. When the MassReg registration is unsuccessful and FRE is high (3.52 mm), the images receive a low similarity score (7.6%) as opposed to a successful registration (0.53–1 mm and 72–99%).

## 4. Conclusions

In this study, we implement and test a new network called MassReg for deformable registration and registration evaluation. We extend existing approaches and DL frameworks to construct this network and propose new techniques for data augmentation and simulation as well as an iterative training approach. With this network in place, registration of DESI and histology images can be performed efficiently and with minimal operator intervention. This should ultimately benefit the development of a DL algorithm for prostate cancer diagnosis with DESI imaging.

Although we validated the architecture on prostate biopsy cores, this strategy can be used on other tissue types and other imaging modalities and is not exclusive to prostate tissue or DESI imaging. Furthermore, with this perioperative system in place, DESI has the potential to provide pertinent information to pathologists and surgeons of the nature of the tissues sampled to further inform diagnosis and adequacy of surgical resections. In the future, this framework can also be extended to other multi-modal image registration problems. Future work may include further investigation into more realistic noise simulation to prevent scoring errors that are influenced by significant background noise.

## Figures and Tables

**Table 1:** Number of image pairs/images used to train each half of the network during each training experiment.

Training Experiment	Train		Test	
	Image Pairs	Total Images	Image Pairs	Total Images
Experiment 1	250	500	8	16
Experiment 2	250	500	8	16
Experiment 3	600	1200	8	16
Experiment 4	500	1000	8	16
Experiment 5	500	1000	8	16

## Data Availability

The data from this study is not available.
